# GLRX3, a novel cancer stem cell-related secretory biomarker of pancreatic ductal adenocarcinoma

**DOI:** 10.1186/s12885-021-08898-y

**Published:** 2021-11-18

**Authors:** Jung Hyun Jo, Sun A Kim, Jeong Hoon Lee, Yu Rang Park, Chanyang Kim, Soo Been Park, Dawoon E. Jung, Hee Seung Lee, Moon Jae Chung, Si Young Song

**Affiliations:** 1 Division of Gastroenterology, Department of Internal Medicine, Yonsei University College of Medicine, 50-1 Yonsei-ro, Seodaemun-gu, Seoul, 03722 South Korea; 2 Institute of Gastroenterology, Yonsei University College of Medicine, Seoul, 03722 South Korea; 3 Cowell Biodigm Co., Ltd, Seoul, South Korea; 4 Department of Biomedical Systems Informatics, Yonsei University College of Medicine, Seoul, 03722 South Korea

**Keywords:** Pancreatic cancer, Cancer stem cell, Biomarker, Glutaredoxin 3, GLRX3, Stemness

## Abstract

**Background:**

Cancer stem cells (CSCs) are implicated in carcinogenesis, cancer progression, and recurrence. Several biomarkers have been described for pancreatic ductal adenocarcinoma (PDAC) CSCs; however, their function and mechanism remain unclear.

**Method:**

In this study, secretome analysis was performed in pancreatic CSC-enriched spheres and control adherent cells for biomarker discovery. Glutaredoxin3 (GLRX3), a novel candidate upregulated in spheres, was evaluated for its function and clinical implication.

**Results:**

PDAC CSC populations, cell lines, patient tissues, and blood samples demonstrated GLRX3 overexpression. In contrast, GLRX3 silencing decreased the in vitro proliferation, migration, clonogenicity, and sphere formation of cells. GLRX3 knockdown also reduced tumor formation and growth in vivo. GLRX3 was found to regulate Met/PI3K/AKT signaling and stemness-related molecules. ELISA results indicated GLRX3 overexpression in the serum of patients with PDAC compared to that in healthy controls. The sensitivity and specificity of GLRX3 for PDAC diagnosis were 80.0 and 100%, respectively. When GLRX3 and CA19–9 were combined, sensitivity was significantly increased to 98.3% compared to that with GLRX3 or CA19–9 alone. High GLRX3 expression was also associated with poor disease-free survival in patients receiving curative surgery.

**Conclusion:**

Overall, these results indicate GLRX3 as a novel diagnostic marker and therapeutic target for PDAC targeting CSCs.

**Supplementary Information:**

The online version contains supplementary material available at 10.1186/s12885-021-08898-y.

## Introduction

Pancreatic ductal adenocarcinoma (PDAC) is known to have poor prognosis with a 5-year survival rate of less than 5%, and radical surgery is the only curative treatment [1]. However, only 10–20% of patients are candidates for surgery at the time of diagnosis. Over the past decade, several cancer-related genes have been identified in PDAC. However, pancreatic cancer remains a disastrous disease with poor prognosis and high frequency of recurrence or metastasis. Thus, accurate and sensitive biomarkers are needed to improve the detection rate of early cancer and the predictability of recurrence after treatment.

Cancer stemness is the integrated functioning of molecular programs that govern and maintain the stem cell state; these cells can be prospectively isolated from the remaining tumor cells and are shown to have clonal long-term repopulation and self-renewal capacity [2]. Cancer cells that can exclusively regenerate tumors have operationally been called cancer stem cells (CSCs). Importantly, CSCs are resistant to radiation and chemotherapeutic drugs. From the first identification of CSCs in myeloid leukemia [3], they have been subsequently identified in solid tumors including PDAC. Several surface markers have been reported for isolating pancreatic CSCs including CD24, CD44, epithelial-specific antigen (ESA), CD133, CXCR4, c-Met, and a combination of these markers [4–7]. Several molecules involved in CSC-related pathways have also been identified. Overall, CSCs have emerged as a new potential target to treat PDAC.

The only biomarker currently recommended for clinical use by the National Comprehensive Cancer Network guidelines for PDAC is carbohydrate antigen 19–9 (CA 19–9) [8]. However, about 10% of the population does not generate this specific sialyl antigen and are thus termed as non-secretors [8, 9]; further, the sensitivity of PDAC detection by using CA 19–9 is about only 75%. The correlation between CA19–9 and the prognosis of patients with PDAC remains controversial. Further, its positive predictive value (PPV) was calculated at 0.9% in an asymptomatic population. A study in Japan screened 10,162 asymptomatic patients and found only 4 (0.04%) cases of PDAC [10]. Based on these data, screening asymptomatic individuals using CA19–9 is not feasible for the early detection of PDAC. Thus, PDAC treatment targets as well as biomarkers for early detection and prognosis prediction after treatment need to be developed.

In the present study, we used the sphere culture method for pancreatic CSC enrichment and analyzed the secretome of pancreatic CSCs compared with that of adherent cells by using two-dimensional gel electrophoresis and MALDI-TOF for biomarker discovery. Several surface marker candidates of pancreatic CSCs or drug targets were obtained. Among them, we investigated Glutaredoxin3 (GLRX3, alternative name; Protein kinase C (PKC)-interacting cousin of thioredoxin [PICOT)) as a potential pancreatic CSCs marker and possible diagnostic and therapeutic target for PDAC. GLRX3 was found to be overexpressed in PDAC CSC populations sorted by sphere formation assay as well as in human blood samples. GLRX3-silenced PDAC cells showed decreased proliferation, migration, clonogenicity, and tumor formation both in vitro and in vivo. Further, GLRX3 regulated c-MET/PI3K/AKT signaling and altered cancer stemness- and epithelial-mesenchymal transition (EMT)-related molecules. Finally, we investigated the serum level of GLRX3 in PDAC patients and in healthy controls to indicate its potential value for PDAC diagnosis and recurrence prediction.

## Materials and methods

### Clinical samples

All clinical samples were obtained from Severance Hospital, Yonsei University Health System. Thirty-two PDAC tissue samples were collected from January 2010 to December 2014. A pathological grading was performed, and the tumor stage of the tissue samples was determined according to the American Joint Committee on Cancer (AJCC) staging system. After pathological evaluation, a tissue microarray (TMA) was generated using cores from tumors and adjacent normal tissue from each specimen. The serum samples of healthy donors, patients with chronic pancreatitis, and PDAC were collected. This study protocol was approved by the Ethical Committee for the Clinical Research of the Institutional Review Board of Severance Hospital, Yonsei University College of Medicine, Seoul, Korea. Informed consents were obtained from patients.

### Sphere and adherent cell culture of pancreatic cancer cell lines

Eight pancreatic cancer cell lines (AsPC-1, BxPC-3, Capan-1, Capan-2, Cfpac-1, HPAC, MiaPaca-2, and Panc-1) were purchased from the American Type Culture Collection (ATCC). A human pancreatic duct epithelial cell line (HPDE) was kindly provided by Dr. Ming-Sound Tsao (University of Toronto, Ontario, Canada). All cells were grown in each conditioned medium and maintained in an atmosphere of 5% CO_2_/95% air at 37 °C.

For enrichment of CSCs, we cultured two pancreatic cancer cell lines, HPAC and CAPAN-1, in sphere conditioned media on ultralow attachment plates for 7 days according to the methods reported in our previous study [11–13] as spheres of HPAC (HS) and spheres of CAPAN-1 (CS). For the controls, HPAC and CAPAN-1 cells were cultured in sphere conditioned media on normal cell culture plates for 7 days as adherent cells of HPAC (HA) and adherent cells of CAPAN-1 (CA). Single cells were cultured in DMEM/F12 medium containing 0.5% FBS (Hyclone, Logan, UT, USA), 0.5% Bovine Albumin serum Fraction V (Gibco, CA, USA), Insulin-Transferrin-Selenium A (Gibco, CA, USA), 10 ng/ml of hEGF (R&D systems, Wiesbaden-Nordenstadt, Germany), 10 ng/ml of hFGF (R&D, Minneapolis, USA), and 10 ng/ml of hLIF (R&D, Minneapolis, USA) at a density of 1 × 10^3^ cells/ml in ultralow attachment plates (Corning, NY, USA) for 7 days. The growth factors were added every 3 days. For secretory protein preparation, the culture medium was changed to serum-free medium at post-sphere culture 5 days, and then cultured for 2 additional days. For confirming the characteristics of these spheres, the expression of genes related to CSCs were investigated by RT-PCR [12]. As previously reported, the expression of genes including those of the Hedgehog, Notch, and Wnt pathway was increased significantly in spheres than in adherent cells. To investigate novel markers for pancreatic CSCs, secretory protein profiles of spheres and adherent cells were analyzed in the respective cultured media .

### siRNA and shRNA transfection

To inhibit the endogenous GLRX3 mRNA expression, human pancreatic cancer cells were transfected with siRNAs by using RNAiMAX reagent (Invitrogen, Carlsbad, California, US) or with shRNAs by using Lipofectamine2000 reagent (Invitrogen, Carlsbad, California, US), according to manufacturer’s instructions; stable knockdown clones were selected using puromycin. Human GLRX3 specific siRNAs were purchased from Invitrogen (Carlsbad, California, US). Their sequences were as follows: siGLRX3-1S, 5′-UGAGGGAGUUCUUUAGCUAACUCUG-3′ and siGLRX3-1AS, 5′-CAGAGUUAGCUAAAGAACUCCCUCA-3′; siGLRX3-2S, 5′-AAGAAUUUCCACCAUCUGCUUGCUG-3′ and siGLRX3-2AS, 5′-CAGCAAGCAGAUGGUGGAAAUUCUU-3′; siGLRX3-3S, 5′-AAACAUAGAGCUGAGGAUAGGUAGG-3′ and siGLRX3-3AS, 5′-CCUACCUAUCCUCAGCUCUAUGUUU-3′. Stealth™ RNAi negative control duplex was used as a negative control. The shRNA-expressing plasmid targeting human GLRX3 and negative control plasmid were purchased from SABiosciences. The human GLRX3 shRNA sequence was 5′-GTGGAAATTCTTCACAAACAT-3′ and control shRNA sequence was 5′-GGAATCTCATTCGATGCATAC-3′. For shRNA transfection, 5 × 10^4^ cells/well of HPAC were seeded onto 6-well plates the day before transfection. Transfection was performed using Lipofectamine2000 reagent according to the manufacturer’s instructions and stable knockdown clones were selected using puromycin. To inhibit the endogenous Met mRNA expression, human pancreatic cancer cells were transfected with siRNAs by using RNAiMAX reagent (Invitrogen, Carlsbad, California, US). Human Met-specific siRNAs were purchased from Santacruz (Dallas, Texas, US). Control siRNA-A (Santacruz, Dallas, Texas, US) was used as the negative control.

### Proteomic analysis

Equal amounts of secretory proteins (1.0 mg) were isoelectrically focused on an 18-cm Immobiline Drystrip pH 3–10 NL (GE Healthcare, Chicago, Illinois, US) and separated on 9–17% SDS-PAGE gels. The gels were stained with Coomassie Brilliant Blue (CBB) solution and scanned using a GS710 scanning densitometer (Bio-Rad, Hemel Hempstead, UK). The gel images were analyzed using Image Master Platinum 5 (GE Healthcare, Chicago, Illinois, US). Spot pairing of each gel image was performed with the control adherent HPAC and CAPAN-1 cells. Group analysis was performed with the gel image of adherent HPAC and CAPAN-1 as group A and the gel image of spheres of HPAC and CAPAN-1 as group B. Spots with a cut-off ratio greater than 2.0-fold were selected. The selected spots were excised manually from the CBB-stained preparative gel, destained, and then digested using trypsin (Promega, Southampton, UK). Tryptic peptides were desalted and purified using a mixture of Poros R2 and Oligo R3, as described previously [14]. The MS spectra of peptides were generated by spectrometric analysis using a 4800 MALDI-TOF/TOF analyzer (Applied Biosystems, Foster City, CA, USA) in the reflectron/delayed extraction mode with an accelerating voltage of 20 kV, with data summed from 500 laser pulses. The spectrum was calibrated against the tryptic auto-digested peaks (m/z 842.5090 and 2211.1046), and monoisotopic peptide masses were obtained using Data Explorer 3.5 (PerSeptive Biosystems, Framingham, Massachusetts, US). A mass range of m/z 800–4000 was used with 1000 shots per spectrum. For MALDI-TOF-MS, GPS 3.1 software (Applied Biosystems, Foster City, California, US) was used for peak generation. MASCOT (Matrix Science, Boston, Massachusetts, US) was used to identify the peptide sequences present in the protein sequence database (NCBI NR) [15].

### Semi-quantitative RT- PCR

The total RNA from cancer cells was extracted using an RNAeasy extraction kit (Qiagen, Hilden, Germany) according to the manufacturer’s instructions. To quantify the relative gene expression level, PCR was performed using β-actin primers as the control. The PCR primers used were GLRX3 sense, 5′-GGGCGGCTGAGGCAGCT-3′; GLRX3 antisense, 5′-GCA GGGGGCAGCATGAGTC-3′; beta-actin sense, 5′-GGCATCCTCACCCTGAAGTA–3′; beta-actin antisense, 5′-GGGGTGTTGAAGGTCTCAAA-3′.

### Immunohistochemistry

Paraformaldehyde-fixed, paraffin-embedded tissue sections (3–5 μm thickness) were deparaffinized in xylene, rehydrated in a graded ethanol series (100–90–80-70-50-30%), and washed with PBS. Endogenous peroxidase was blocked by immersing the slides in 0.3% (v/v) hydrogen peroxide in methanol for 15 min at room temperature. Microwave antigen retrieval was performed in citrate buffer (0.01 M, pH 6.0). The sections were blocked by soaking in 10% (v/v) normal donkey serum for 1 h, and were then incubated overnight with the primary antibody, anti-human GLRX3 (1:150, Sigma-Aldrich, Inc., St. Louis, MO, US) at 4 °C. The sections were incubated with EnVision/HPR, Rabbit/Mouse (DakoCytomation, CA, US) and diaminobenzidine (DAB+) chromogen. The sections were counterstained with hematoxylin (Sigma-Aldrich, Inc., St. Louis, MO, US), dehydrated, and mounted. Immunoreactivity was scored as a percentage of GLRX3-positive tumor cells– no expression: 0, < 20%: 1+, 20–50%: 2+, and > 50%: 3 + .

### ELISA

Serum GLRX3 and CA19–9 levels in healthy and PDAC patients were measured using ELISA. The ELISA kit for GLRX3 was purchased from USCNK (Wuhan, China). For comparison, serum CA19–9 levels were measured using a commercial immunochemiluminescence kit (VITROS® ECiQ Immunodiagnostic System, Ortho Clinical Diagnostics). All assays were performed according to the manufacturers’ instructions and were proceeded by duplication per sample.

### Soft agar colony formation assay

Five hundred single cells in suspension containing 0.3% agar medium were overlaid on 0.6% agar medium in a 24-well plate (SPL). Each well was covered with complete medium, and the plates were incubated for 4 weeks. The colonies were stained with crystal violet and counted. The experiment was performed in triplicate.

### Flow cytometry and cell sorting

Cultured cells were detached using Accutase solution (Sigma Aldrich, Sigma-Aldrich, Inc., St. Louis, MO, US) and were washed in PBS with 0.5% FBS. Single cells were stained for 20 min on ice in the dark, washed twice in PBS with 0.5% FBS, and then fixed in 2% paraformaldehyde. Flow cytometric analysis was performed on a FACSCalibur system (BS Biosciences, San Jose, CA, US), and cell sorting was performed using FACSAria II (BD Immunocytochemistry System, Franklin Lakes, NJ, US). Antibodies against CD44 (anti-CD44-FITC, BD Pharmingen, Franklin Lakes, USA) and c-Met (anti-c-Met-FITC, eBioscience, San Diego, California, US) were used. Antibodies for cell sorting against CD24 (anti-CD24-PE, BD), CD44 (anti-CD44-APC, BD), and ESA (anti-ESA-FITC, BD) were used. FITC-mouse IgG2b, κ isotype control (BD), rat IgG1 κ isotype control FITC (eBioscience, San Diego, California, US), PE-mouse IgG2a, κ isotype control (BD Immunocytochemistry System, Franklin Lakes, NJ, US), and APC-mouse IgG2b, κ isotype control (BD Immunocytochemistry System, Franklin Lakes, NJ, US) were used as the controls.

### Protein extraction and western blot

Cells were prepared in lysis buffer containing 50 mM HEPES (pH 7.2), 150 mM NaCl, 25 mM beta-glycerophosphate, 25 mM NaF, 5 mM EGTA, 1 mM EDTA, 1% NP-40, 1 mM sodium orthovanadate, 0.1 mM PMSF, and a Protease Inhibitor cocktail (leupeptin, pepstatin, aprotinin, and antipain; each 5 μg/ml). For secretory protein preparation, the culture medium was centrifuged, and cellular components and debris were discarded. The culture medium was concentrated using 10 K cut-off microcon (Amicon), or by adding ice-cold acetone, the precipitated protein was resuspended in lysis buffer. The proteins were separated on SDS-PAGE and transferred to a 0.45-μm Immobilon P-transfer membrane (Millipore). The membrane was blocked in 5% (w/v) non-fat milk and then probed with a primary antibody; anti-human GLRX3 antibody, beta-catenin, E-cadherin, GAPDH (Santacruz, Dallas, Texas, US), c-MET, PI3K, pAKT (Cell signaling, Danvers, Massachusetts, US), AKT, Wnt1, 3, 5a, 7b,11, 16, RhoA, RhoB, pJNK, RAC1, Dvl2 (Santacruz, Dallas, Texas, US), and ABCG2 (Abcam, Cambridge, UK). The immunoreactive material was then visualized using SuperSignal West Pico Chemiluminescent substrate (Pierce Biotechnology, Rockford, Illinois, US) according to the manufacturer’s instructions.

### In vivo tumorigenesis

Cells were suspended with 50% Matrigel (BD biosciences) in HBSS (Invitrogen Inc.) to a final count of 3 × 10^7^/ ml. Then, 200 μL of the cell suspension was injected subcutaneously into 6-week-old male NOD/SCID or nude mice. Tumor formation was monitored twice a week. Tumor volumes were calculated using the formula V (mm^3^) = A × B^2^, where A is the largest dimension, and B is the perpendicular diameter. After 14 weeks, tumor xenografts were recovered from the mice, fixed in 4% paraformaldehyde, and embedded in paraffin. Experimets were approved by The Institutional Animal Care and use committee (IACUC) of Yonsei University College of medicine based on the animal protection act (Approval number: 2010–0294).

### Growth rate and MTT assay

Cells were seeded at 2 × 10^3^ cells/well into 24-well plates, and the number of cells was counted every 24 h. The experiment was performed in triplicate to determine the number of cells at each time point. After incubation at 37 °C overnight, the cells were treated with various concentrations of gemcitabine in complete growth media and then incubated for 72 h at 37 °C. A 3-(4,5-dimenthelthiazol-2-ly)-2,5-diphenyltetrazolium bromide-based assay (absorbance 570 nm) was used to measure the number of metabolically active cells.

### Statistical analysis

Serum GLRX3 and CA19–9 levels were compared between normal and pancreatic cancer patients, by using the Kruskal-Wallis test, which is a non-parametric statistical test. Cox regression, cut-off value, receiver operating characteristic (ROC) curve, area under the ROC curve (AUC), and 95% confidence intervals (CI) were determined using SAS, version 9.2 (SAS Institute Inc., Cary, NC, USA) and the R package, version 3.4.1 (http://www.R-project.org). All data were expressed as the mean ± standard error of the mean (SEM) or the median ± standard deviation (SD).

We downloaded the raw RNAseq data level 3 and quantified the transcript models using RNA-Seq Expectation Maximization (RSEM)15 from the Broad Firehose TCGA pipeline, GDAC Firehose.16. RNAseq preprocessing was performed using the R package, EdgeR (version 3.40.6) [16] and Limma (version 3.26.8) [17]. Statistical evaluation for TCGA datasets was carried out using R, version 3.4.1 (http://www.r-project.org). GLRX3 and other gene expression levels in pancreatic cancers were compared using TCGA dataset and Pearson correlation. Outlier samples in the total population were identified using quantiles, and samples from the upper and lower 0.1 quantiles were removed from the actual analysis. The patients’ clinical outcome data were analyzed using the survival package and were plotted as Kaplan–Meier survival curves. Unless specified otherwise, *p* values smaller than 0.05 were considered significant. An exact p value was calculated where applicable.

Clinical data from patients’ samples were analyzed using the χ2 and Fisher exact tests for categorical data and the Student’s *t* test and Mann-Whitney U test for continuous variables. Multivariate analysis was performed to evaluate the possible significant factors, considering the influence of confounding clinical variables. Hazard ratios (HRs), 95% confidence intervals (95% CIs), and *p* values of multivariate analysis were calculated using a Cox proportional hazards model for OS by using variables that were statistically significant. Overall survival was estimated and compared using the Kaplan-Meier analysis with a log-rank test. All statistical analyses were performed using IBM SPSS Statistics for Windows, version 25.0 (IBM Corp, Armonk, NY). *p* < 0.05 was considered statistically significant.

## Results

### Comparison of secretory protein profiles between spheres and adherent cells

Additional file 1: Fig. S1 shows 2D gel images for the secretory proteins extracted from spheres and adherent cells of HPAC and CAPAN-1: spheres of HPAC (HS), spheres of CAPAN-1 (CS), adherent cells of HPAC (HA), and adherent cells of CAPAN-1 (CA). In total, 626, 576, 642, and 515 spots were obtained in the culture media from HS, HA, CS, and CA cells, respectively, and 587 spots across the four gels were matched.

To compare the two spheres and adherent cells, gel images of HA and CA were classified as group A and gel images of HS and CS were classified as group B. As a result, 200 spots of group B including 55 increases and 145 decreases were differentially expressed by at least two-fold compared with those in the control group A. For identifying the differentially expressed proteins, 55 upregulated spots in spheres were further subjected to MALDI-TOF analysis. In total, 53 spots were identified to 46 proteins and these upregulated proteins in spheres compared to adherent cells are listed in Additional file 1: Table S1. All proteins were analyzed using secretomeP 2.0 and SignalP 4.1 to predict their secretory potential. About 52% of 46 proteins were found to be potentially secreted through classical or non-classical secretion pathways.

Among them, a list of the proteins associated with CSCs or reported as targets for various cancers including PDAC is presented in Table 1. Heat shock protein 27 (spot no. 16193) has been reported as a potential serum marker and to cause gemcitabine resistance in PDAC [18–22]; further, overexpression of neutrophil gelatinase-associated lipocalin (spot no. 16663 and 16,246) has been reported in transgenic PDAC mouse model and in serum from a patient with PDAC as a potential biomarker [23, 24]. Furthermore, 35 proteins were previously reported as upregulated in cancers including PDAC, and 19 proteins were reported to be correlated with CSCs. HSP90 (spot no. 15391 and 15,602), Grp78 (spot no. 15413), Grp94 (spot no. 15538), and HSP27 (spot no. 16193), belonging to the HSP family, have been reported as therapeutic targets for PDAC [14, 25, 26]. Overexpression of Aldo-keto reductase proteins, AKR1B1 (spot no. 15977, 15,978, and 16,000) and AKR1C2 (spot no. 15965), has also been observed in various cancer tissues including PDAC [27–29]. Proteins involved in tumor metastasis and invasion, including cathepsin D (spot no. 16095), vimentin (spot no. 15687) and keratin 9 (spot no. 16276) [30, 31], were also overexpressed in spheres compared to adherent cells. **KRAS mutation is a hallmark of PDAC. PIK3CA (spot no. 16432), a downstream effector of RAS and mutant of PIK3CA, has been reported in in breast, ovarian, and colorectal cancer, and coexists with RAS (KRAS and NRAS) and BRAF mutations** **[**32**–**35**].** ALDH (spot no. 16610) was also increased in the spheres, consistent with increased ALDH activity in the serum of patients with PDAC; ALDH activity is also enhanced in the tumor-initiating population related to CD133 or CD44 and contributes to chemoresistance and radiation resistance in pancreatic cancer, breast cancer, and lung cancer [36–39]. Transferrin (spot no. 16676) is used to supplement the sphere culture with iron, and its receptor, transferrin receptor, has been reported as a potential diagnostic and therapeutic target for PDAC [40, 41]. Prominin-1/CD133 has also been reported as a pancreatic CSC marker; further, CD133 has been reported as important in transferrin uptake through the CD133-Tf-iron network [42]. These data indicate that our proteomic results have strong reliability for searching novel secreted protein candidates in pancreatic CSCs. Among these proteins, we selected GLRX3 for further investigation.

**Table 1:** List of proteins associated with cancer stem cells that were upregulated in spheres compared to adherent cells of pancreatic cancer cell lines

Spot number	Protein Identified
15,391 and 15,602	heat shock protein 90 (HSP90)
15,413	glucose regulated protein 78 (GRP78)
15,538	glucose regulated protein 94 (GRP94)
15,687	Vimentin
15,890	Glutaredoxin3 (GLRX3)
15,977, 15,978 and 16,000	aldo-keto reductase family 1 member B1 (AKR1B1)
15,965	aldo-keto reductase family 1 member C2 (AKR1C2)
16,095	Cathepsin D
16,193	heat shock protein 27 (HSP27)
16,276	keratin 9
16,432	PIK3CA
16,610	aldehyde dehydrogenase (ALDH)
16,663 and 16,246	neutrophil gelatinase associated lipocalin
16,676	Transferrin

### GLRX3 is highly expressed in cultured pancreatic cells and in enriched CSCs

**Fig. 1:**
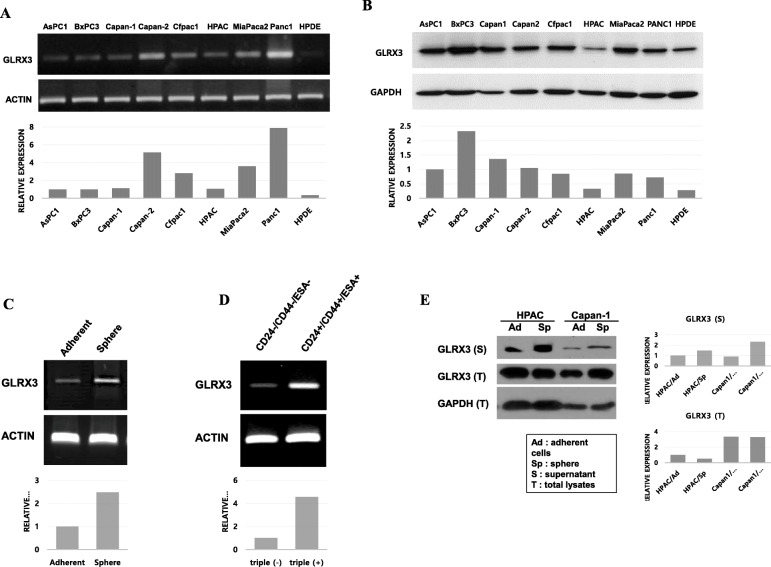
Validation GLRX3 expression in pancreatic cancer cell lines: **A**-**B** GLRX3 expression in pancreatic cancer cell lines. **A** GLRX3 mRNA was expressed in various pancreatic cancer cell lines. **B** GLRX3 protein was expressed in various pancreatic cancer cell lines. **C** Semi-quantitative RT-PCR of GLRX3 levels in adherent cells and spheres. GLRX3 mRNA was overexpressed in spheres compared to adherent HPAC cells; **D** Semi-quantitative RT-PCR of GLRX3 levels in CD24+/CD44+/ESA+ cells and CD24−/CD44−/ESA- cells. HPAC cells were fluorescence stained with CD24-PE, CD44-APC, and ESA-FITC, and then isolated by FACS. GLRX3 mRNA was increased in CD24+/CD44+/ESA+ cells than in the CD24−/CD44−/ESA- cells; **E** Confirmation of GLRX3 in western blot analysis. GLRX3 showed increased expression in the supernatant of spheres compared to that of adherent cells. Between total lysate of spheres and adherent cells, GLRX3 showed similar expressions in HPAC and increased expression in spheres of the Capan-1

To investigate the level of GLRX3 expression in human pancreatic cancer cell lines, we performed semi-quantitative RT-PCR and western blot analysis by using various cell lines. GLRX3 mRNA was expressed in various PDAC cell lines (Fig. 1A). Western blot analysis showed that GLRX3 protein was also expressed in various PDAC cell lines (Fig. 1B). HPDE cell line, normal pancreatic duct cells, expressed a relatively lower level of GLRX3 compared to cancer cell lines.

Next, overexpression of GLRX3 mRNA in spheres compared to that in adherent cells was confirmed by semi-quantitative PCR (Fig. 1C). Therefore, we hypothesized that GLRX3 may play a functional role in maintaining self-renewal or stem-like properties in pancreatic cancer. However, the reported CSC markers were varied and partially overlapped with other populations [43]. Therefore, we verified whether GLRX3 was overexpressed in other pancreatic CSC populations. The CD24+/CD44+/ESA+ cells are well known as a pancreatic CSC population [4]. Therefore, we isolated these CSCs by using the combination of triple-positive CD24, CD44, and ESA cell surface markers from HPAC cells by fluorescence-activated cell sorting (FACS), and then performed semi-quantitative PCR to measure GLRX3 expression in CSCs (CD24+/CD44+/ESA+) and in surface marker negative cancer cells (CD24−/CD44−/ESA-) [44]. We found that GLRX3 mRNA was overexpressed in CD24+/CD44+/ESA+ cells compared to CD24−/CD44−/ESA- cells (Fig. 1D). These data suggest that GLRX3 may also be overexpressed in other pancreatic CSC populations.

GLRX3 protein expression was found to be increased in the culture media of spheres than in adherent cells. Between total lysate of spheres and adherent cells, GLRX3 showed similar expressions in HPAC and increased expression in spheres of the Capan-1 (Fig. 1E). This result reflected the GLRX3 had a certain role in CSC as a secretory protein.

### GLRX3 is highly expressed in samples from patients with PDAC

**Fig. 2:**
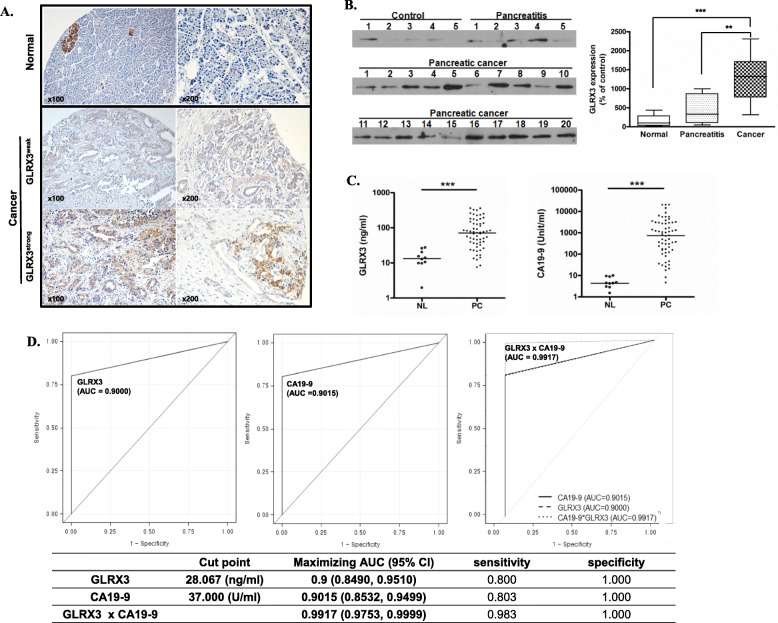
GLRX3 is overexpressed in the tissues and blood from patients with pancreatic cancer: **A** GLRX3 is overexpressed in pancreatic cancer tissues. Immunohistochemical staining was performed on a pancreatic tissue microarray. Representative images show islet cells expressing GLRX3 in normal pancreas tissues and its overexpression in adenocarcinoma; **B** Western blot analysis was performed in depleted plasma from healthy individuals (*n* = 5), patients with chronic pancreatitis (*n* = 5), and patients with pancreatic cancer (*n* = 20). GLRX3 was found to be overexpressed in the plasma of patients with pancreatic cancer compared to that in plasma from healthy individuals (*p* < 0.001) and patients with chronic pancreatitis (*p* = 0.005). Values of GLRX3 expression were estimated using an image analysis system (BAS2500, Fujifilm, Tokyo, Japan) and were normalized to mean value of the control group (regarded as 100%); **C** Dot plot for the serum level of GLRX3 and CA19–9 by ELISA. The horizontal line represents the median. The serum levels of GLRX3 and CA19–9 were significantly different between patients with pancreatic cancer and healthy persons (All *p* < 0.001); **D** ROC curves of patients with pancreatic cancer versus healthy persons for GLRX3, CA19–9, and their mathematical combination. When GLRX3 and CA19–9 were combined, the AUC was increased compared to that with GLRX3 or CA19–9 alone (*p* < 0.0001)

To determine the expression of GLRX3 in human PDAC tissues, we performed immunohistochemical staining for GLRX3 in a pancreatic tissue microarray (TMA). Immunohistochemical staining revealed strong cytoplasmic expression of GLRX3 in cancer cells (Fig. 2A). The islet cells in normal tissue also showed positive immunoreactivity. However, normal pancreatic ducts and acinar cells did not react with the GLRX3 antibody. Additional file 1: Table S2 presents the clinical characteristics of the patients. Of 32 cases, 20 (62.5%) showed positive GLRX3 expression in cancer tissues. The mean CA 19–9 level at the time of diagnosis was 276.6 ng/ml. The overall survival (OS) was 17.6 months. The tumor, nodes, and metastasis (TNM) stages were confirmed by pathology reports after surgery. Thirty-one patients (96.9%) were staged T3, and one (3.1%) was staged T2. Twenty patients were classified as N0 (62.5%), and 45 were classified as stage N1 (37.5%). The results of comparative analysis between the GLRX3-negative and GLRX3-positive groups are presented in Additional file 1: Table S2. There were no significant differences between groups in aspect of clinical characteristics. The disease-free survival (DFS) and OS were shorter in the GLRX3-positive group (15.4 vs. 9.0 months for DFS, 21.5 vs. 13.9 months for OS); however, the differences were not statistically significant.

To confirm the potential of GLRX3 as a secretory biomarker for pancreatic tumor initiating cells, we examined GLRX3 in patient plasma samples by western blot analysis. To eliminate the six highly abundant proteins (albumin, transferrin, IgG, IgA, haptoglobin, and anti-trypsin) in plasma, we used a multiple affinity removal column system (MARS) [45]. Plasma samples from five healthy individuals, five patients with chronic pancreatitis, and twenty patients with pancreatic cancer were used for the western blot analysis (Additional file 1: Table S3). As shown in Fig. 2B, GLRX3 expression was increased in the plasma of patients with pancreatic cancer compared to that in the plasma of normal individuals or patients with chronic pancreatitis. The expression levels of GLRX3 in pancreatic cancer were 8.8-fold greater than those in control plasma (*p* < 0.001) and 2.8-fold greater than those in chronic pancreatitis (*p* = 0.005). These data confirmed GLRX3 as a secretory biomarker protein detectable in human blood that is upregulated in the plasma of patients with pancreatic cancer than in healthy individuals or in chronic pancreatitis.

### GLRX3 is a potential diagnostic marker for PDAC

To evaluate the diagnostic significance of GLRX3 compared with CA19–9, we examined GLRX3 by enzyme-linked immunosorbent assay (ELISA) in 70 individual serum samples from normal healthy individuals (*n* = 10; 6 males and 4 females; median age 47.5-year old with range 40 ~ 63) and from patients with pancreatic cancer (*n* = 60, details in Additional file 1: Table S4). Serum samples were used for the ELISA, as the commercial ELISA kit was more sensitive to serum than to plasma. The median serum levels of GLRX3 in normal conditions and in pancreatic cancer were 13.27 ng/ml (range; 1.94–27.18 ng/ml) and 70.84 ng/ml (range; 7.5–357.64 ng/ml), respectively, with a significant difference (*p* < 0.0001) (Fig. 2C). The median serum levels of CA19–9 in normal conditions and in pancreatic cancer were 7 (0.9–21.5 U/ml) and 491.5 (4–20,000 U/ml), respectively (p < 0.0001) (Fig. 2C). To evaluate the potential of serum GLRX3 and CA19–9 levels to differentiate between normal and pancreatic cancer samples, we calculated the area under the curve (AUC) by using a receiver operating characteristic (ROC) curve. For GLRX3, the AUC was 0.9000 (95% CI: 0.8490, 0.9510), and that of CA19–9 was 0.9015 (95% CI: 0.8532, 0.9499), without a significant difference (*p* = 0.3462) (Fig. 2D). With the best cut-off value of 28.067 ng/ml, the sensitivity and specificity of GLRX3 to differentiate pancreatic cancer from normal conditions were 80.0 and 100%. For the CA19–9, the sensitivity and specificity were 80.3 and 100% at a cut off value of 37 U/ml. For the best diagnostic marker combination, we combined GLRX3 and CA19–9. When GLRX3 and CA19–9 were combined, the area under the curve (0.9917: 95% CI: 0.9753, 0.9999) increased further compared to that with GLRX3 or CA19–9 alone (All *p* < 0.0001) (Fig. 2D). These results indicate that GLRX3 alone or in combination with CA19–9 could be a potential diagnostic biomarker for pancreatic cancer.

Next, the correlation of plasma GLRX3 levels with patient survival were evaluated among patients with pancreatic cancer. As shown in Table 2, multivariate Cox-hazard proportional analysis showed that high serum GLRX3 levels were significantly associated with disease free survival (DFS) after surgery (Hazard ratio 1.009, 95% CI 1.002–1.016, *p* = 0.008). With the best cutoff value (40 ng/mL) of serum GLRX3 levels for survival analysis calculated using the Log-rank test, high GLRX3 levels (*n* = 13, DFS 7.7 months, 95% CI 5.4–10.0) were associated with poor DFS in patients compared those with low GLRX3 levels (*n* = 7, DFS 13.0 months, 95% CI 9.0–17.1, *p* = 0.041 by the Log-rank test). This result suggested that a high serum GLRX3 level at diagnosis can be a risk factor for PDAC recurrence after surgery. However, progression free survival (PFS) after palliative chemotherapy and overall survival (OS) did not show any association with serum GLRX3 levels (Table 2B, C).

**Table 2:** Multivariate Cox-proportional hazard analysis for the contribution of clinical factors to DFS, PFS, and OS

	Univariate		Multivariate	
	Hazard ratio (95% CI)	*p* value	Hazard ratio (95% CI)	*p* value
**A. DFS after surgery (*n*** **= 20)**				
High GLRX3	1.007 (1.001–1.013)	0.027	1.009 (1.002–1.016)	0.008
Older age	0.975 (0.934–1.018)	0.253	0.976 (0.933–1.022)	0.306
Male gender	0.393 (0.125–1.241)	0.111	0.274 (0.081–0.932)	0.038
Higher CA19–9	1.000 (0.999–1.000)	0.742	1.000 (0.999–1.000)	0.305
**B. PFS after palliative chemotherapy (*n*** **= 47)**				
High GLRX3	1.001 (0.997–1.005)	0.569	1.001 (0.997–1.005)	0.569
Older age	1.014 (0.988–1.041)	0.301	1.011 (0.986–1.037)	0.379
Male gender	0.589 (0.320–1.087)	0.090	0.589 (0.320–1.087)	0.090
Higher CA19–9	1.000 (1.000–1.000)	0.361	1.000 (1.000–1.000)	0.192
**C. OS after initial diagnosis (*n*** **= 60)**				
High GLRX3	1.001 (0.998–1.004)	0.451	1.001 (0.998–1.004)	0.619
Older age	1.013 (0.988–1.039)	0.320	1.012 (0.987–1.038)	0.347
Male gender	0.611 (0.343–1.091)	0.096	0.611 (0.343–0.932)	1.091
Higher CA19–9	1.000 (1.000–1.000)	0.313	1.000 (1.000–1.000)	0.401

*Abbreviations: DFS* disease-free survival*, PFS* progression-free survival*, OS* overall survival*, CI* confidence interval

### Effect of shRNA-mediated GLRX3 knockdown in pancreatic cancer cells

**Fig. 3:**
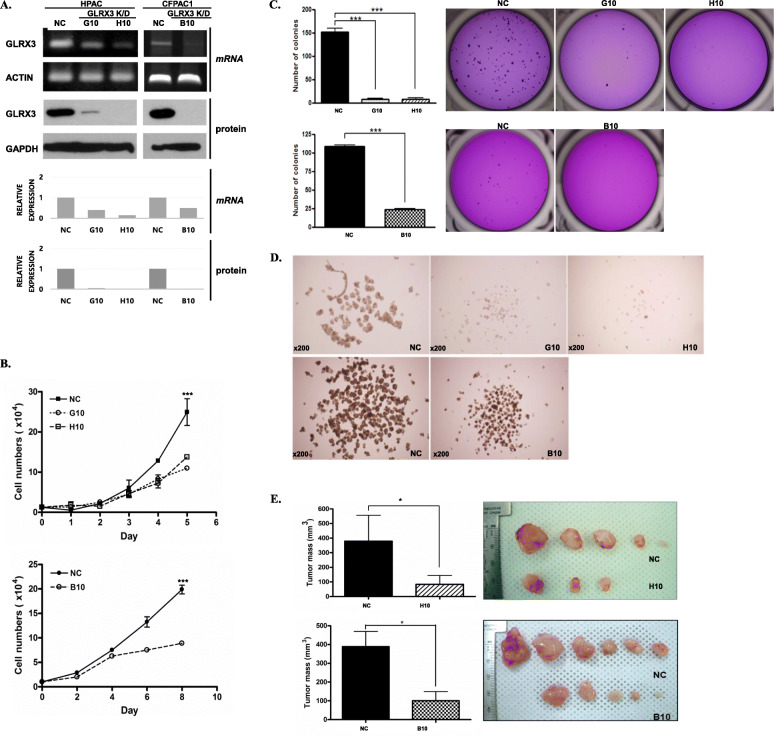
Effect of GLRX3 inhibition in HPAC and CFPAC-1 pancreatic cancer cells: **A** Establishment of GLRX3 knockdown cells in HPAC and CFPAC-1 pancreatic cancer cell lines. GLRX3 mRNA and protein levels were downregulated by shRNA transfection. Β-actin and GAPDH served as loading controls; **B** Cell proliferation was reduced in shGLRX3 transfected cells. The transfected cells (2 × 10^3^ cells/well) were counted every 24 h using a hemocytometer. The experiment was performed in triplicate and the data are shown as the mean ± SEM; **C** Colony formation was reduced by GLRX3 knockdown. shControl and shGLRX3 cells were cultured on agar media for 4 weeks. The experiment was performed in triplicate and data are shown as the mean ± SD (*p* < 0.001). Representative images (0.8x) and graphs were obtained at the end of the experiment; **D** Formation of spheres was reduced by GLRX3 knockdown. The shControl and shGLRX3 cells (1 × 10^3^ cells/ml) were cultured in sphere conditioned media on ultralow attachment plates for 7 days. Representative 4x photomicroscope images showed the spheres at 7 days after culture; **E** shGLRX3 cells formed no or smaller tumors than the shControl cells in vivo. The shGLRX3 and shControl cells were injected into the flank of 6-week old male SCID (HPAC NC and H10 clone) or nude (CFPAC-1 NC and 3B10 clones) mice (*n* = 5/group) and monitored for 14 weeks. Representative graft images show the results of tumor xenografts at the end of the experiments

To determine the role of GLRX3 in pancreatic cancer cells, shRNA targeting human GLRX3 (shGLRX3) or the control vector (shControl) were stably transfected into HPAC and CFPAC-1 cells and were selected using puromycin. Selected clones of shGLRX3 transformed cells (G10 and H10; HPAC, B10; CFPAC-1) expressed similar levels of downregulated GLRX3. The mRNA and protein levels of GLRX3 were reduced in G10, H10, and B10 cells, compared to the control shRNA-transfected cells (NC) (Fig. 3A).

The biological function of GLRX3 was evaluated by comparing cell growth between the control and GLRX3 knockdown cells. Cell proliferation was reduced in shGLRX3 cells than in control cells (Fig. 3B). The transfected cells (2 × 10^3^ cells per well) were counted every 24 h by using a hemocytometer. The experiment was performed in triplicate and the data are shown as the mean ± SEM in Fig. 3B; the cell numbers at day 5 were significantly decreased in G10 and H10 cells similar to those at day 8 in B10 cells compared to the control cells (All *p* < 0.001). Moreover, colony formation was inhibited significantly in GLRX3 knockdown cells compared to the control cells in soft agar (Fig. 3C, mean ± SD, H10; 8.000 ± 2.944 vs. G10; 25.50 ± 3.873 vs. NC; 152 ± 7.874 in HPAC, B10; 25.33 ± 1.155 vs. NC; 108.3 ± 2.082 in CFPAC1, All p < 0.001). To evaluate the role of GLRX3 in pancreatic CSC self-renewal and long-term growth potential, we performed an in vitro tumorsphere assay and colony-forming assay by using GLRX3 knockdown and control HPAC cells. As a result, GLRX3 knockdown cells did not form tumorspheres, whereas control cells formed tumorspheres (Fig. 3D). To verify the function of GLRX3 in pancreatic cancer tumorigenicity in vivo, we injected GLRX3 knockdown or control cells subcutaneously into SCID mice and measured the resulting tumor growth after 14 weeks (Fig. 3E). The GLRX3 knockdown H10 clone from HPAC and B10 clone from CFPAC-1 cells showed tumor formation in only 60% (3/5) and 83.3% (5/6) of the mice, whereas the control cells showed tumor formation in 100% (5/5 and 6/6). Moreover, the tumors derived from H10 GLRX3 knockdown cells, were 78% smaller than those derived from control cells (mean ± SEM; 83.6 ± 60.9 mm^3^ vs. 379.9 ± 176.2 mm^3^), and G10 were 74% smaller than control cells (mean ± SEM; 100.6 ± 120.5 mm^3^ vs. 388.9 ± 197.7 mm^3^). These results suggest that GLRX3 is involved in the self-renewal and long-term survival of pancreatic CSCs.

### Regulation of c-met signaling in PDAC cells by GLRX3

**Fig. 4:**
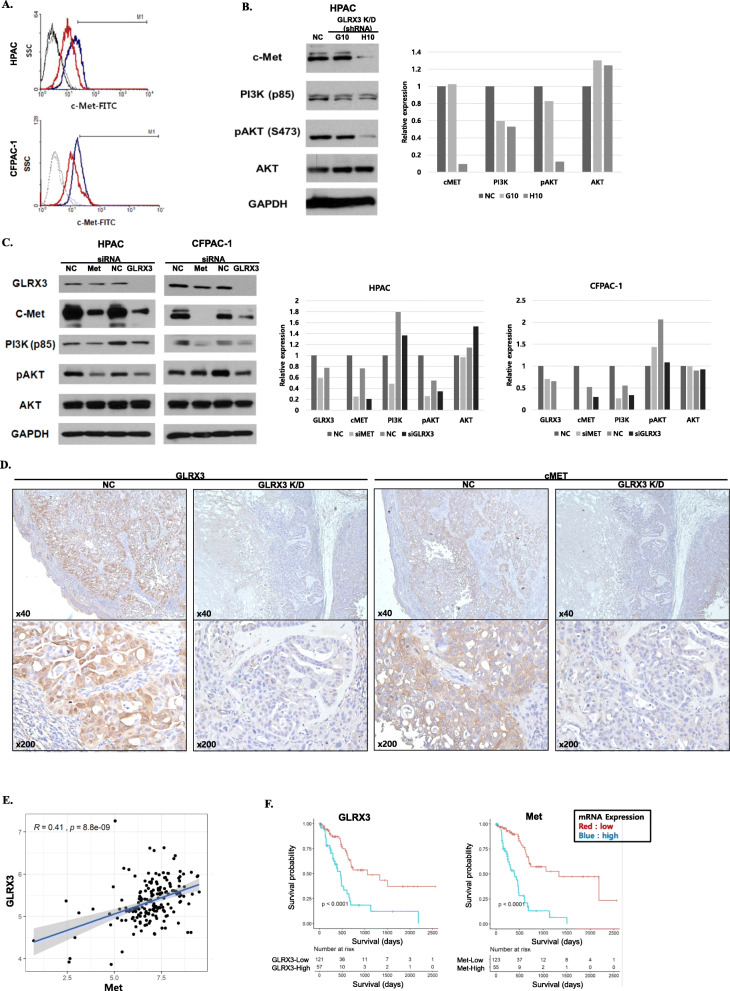
GLRX3 regulates c-Met signaling in PDAC cells: **A** Flow cytometry analysis of c-Met expression. Histograms indicate c-Met-FITC positive cells. c-Met positive cells were reduced upon GLRX3 knockdown (red) compared to the control (blue); **B** In western blot analysis, c-MET and its downstream signal molecules were downregulated by shRNA transfection in HPAC cells; **C** The effect of GLRX3 or Met silencing on Met/PI3k/AKT singling in HPAC cells. The expression of c-Met, PI3K, and phosphorylation of AKT was reduced by siRNA transfection; **D** Immunohistochemical analysis of mouse tumor tissues from CFPAC-1 NC and GLRX3 K/D cells (3B10 clones) revealed cMET expression was significantly decreased in GLRX3 K/D tumor than in control; **E** Glrx3 and Met mRNA expression levels in pancreatic cancer tissues were downloaded from the TCGA by using ISB Cancer Genomics Cloud. Outlier samples were removed by clipping the top and bottom deciles of each expression distribution. The correlation between GLRX3 and Met mRNA expression is illustrated as a scatter plot where each dot represents a single cancer tissue sample. The Pearson’s correlation value (R), and the *p* value are indicated; **F** The Glrx3 and Met mRNA expression and cancer patients’ clinical data were derived from the TCGA database. Patients were divided into low (red line) or high (blue line) expression groups, using maximally selected rank statistics. Survival of patients was visualized using the Kaplan–Meier plot. The log-rank p value between the groups are shown in each plot

The expression of known pancreatic CSC markers was documented by flow cytometry. As shown in Fig. 4A, c-Met expression was reduced to 5.70 ± 3.30% (mean ± SD) in GLRX3 knockdown HPAC cells, compared with 23.81 ± 3.35% in control cells. In GLRX3 knockdown CFPAC-1 cells, c-Met was reduced to 17.32 ± 0.89%, compared with 33.87 ± 0.60% in control cells. c-Met is a well-known CSC marker and c-Met-high cells are reported to demonstrate tumorigenicity in vivo [46]. These results suggest GLRX3 may be involved in the c-Met signaling pathway.

To evaluate whether there were any changes in the c-Met level and its downstream signaling by GLRX3 knockdown, we assayed the c-Met downstream signal molecules by western blot analysis. The results showed that c-Met, PI3K, and phosphorylation of AKT were reduced in GLRX3 knockdown HPAC cells (G10 and H10 cell lines) (Fig. 4B). These results indicate that GLRX3 is involved in the Met/PI3K/AKT pathway. To further evaluate the relation between Met and GLRX3, we assayed the c-met downstream signal molecules by western blot analysis in HPAC and CFPAC-1 cell lines subjected to siRNA-mediated Met or GLRX3 knockdown (Fig. 4C). GLRX3 knockdown cell lines showed downregulation of c-Met, PI3K, and phosphorylation of AKT compared to the siRNA control transfected cell lines. However, siRNA targeting Met knockdown reduced the expression level of c-Met, PI3K, phosphorylation of AKT, but not GLRX3. In addition, immunohistochemical analysis of mouse tumor tissues from CFPAC-1 NC and GLRX3 K/D cells (3B10 clones) revealed cMET expression was significantly decreased in GLRX3 K/D tumor than in control (Fig. 4D).

These results indicate that GLRX3 downregulation correlates with downregulation of the c-MET signaling pathway, suggesting that GLRX3 gene transcription and translation may correlate with MET mRNA expression. To analyze whether such a correlation might exist, we downloaded the data on GLRX3 and MET mRNA expression levels in human pancreatic cancers available at The Cancer Genome Atlas (TCGA). Using the Pearson’s correlation coefficient, we examined the extent of correlation between GLRX3 and MET, after clipping the top and bottom decile of each measurement to avoid any outlier effects (Fig. 4E). A general tendency of positive correlation was observed between the GLRX3 and MET mRNA expression levels (R = 0.41, *p* < 0.0001). Figure 4F demonstrates pancreatic cancer patients’ survival analysis by using TCGA datasets according to GLRX3 and MET expression as visualized by the Kaplan-Meier plot. The patients were divided into two groups, high and low, based on the mRNA expression levels by maximally selected rank statistics with a threshold between 20 and 80%, respectively. GLRX3 high expressers and MET mRNA high expressers presented decreased overall survival compared to the low expressers (All p < 0.0001 by Log-rank test). The Pearson’s correlation coefficient between GLRX3 mRNA and the mRNA of other c-MET pathway molecules such as PIK3CA, PIK3CD, and AKT1, and the survival curves according to the mRNA expression levels, are demonstrated in Additional file 1: Fig. S2 and S3.

### Effect of GLRX3 knockdown on cancer stemness-related molecules

**Fig. 5:**
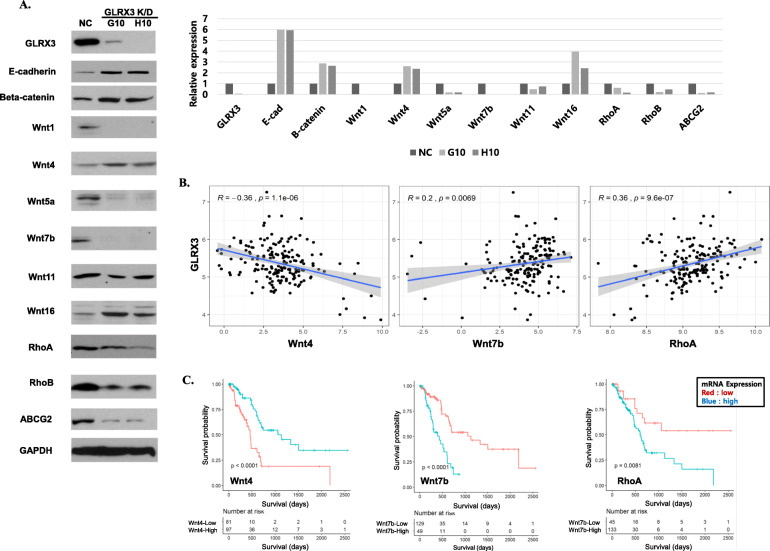
Effect of GLRX3 knockdown in cancer stemness-related molecules in pancreatic cancer cells and the mRNA expression in TCGA data: **A** After shRNA-mediated GLRX3 knockdown in HPAC cells, GLRX3-silenced cell lines (G10 and H10) presented altered expression of EMT-related proteins. E-cadherin and beta-catenin were upregulated and Wnt family, RhoA, RhoB and ABCG2 presented altered expression compared to the control cells; **B**-**C** showed the representative results of well-correlated TCGA data analyses with GLRX3 K/D cell line studies. **B** GLRX3 and stemness related gene expression levels in pancreatic cancer tissues were downloaded from the TCGA by using ISB Cancer Genomics Cloud. Outlier samples were removed by clipping the top and bottom deciles of each expression distribution. The correlation between GLRX3 and representative gene mRNA expression is illustrated as a scatter plot where each dot represents a single cancer tissue sample. The Pearson’s correlation value (R), and the p value are indicated; **C** GLRX3 and stemness related gene mRNA expression and cancer patients’ clinical data were derived from the TCGA database. Patients were divided into low (red line) or high (blue line) expression groups, using maximally selected rank statistics. Survival of patients was visualized using Kaplan–Meier plots. The log-rank *p* values between the groups are shown in each plot

As shown in Fig. 5A, E-cadherin, an epithelial marker of epithelial mesenchymal transition (EMT) was induced in GLRX3 knockdown cells (G10 and H10; GLRX3 shRNA transfected HPAC cell lines) compared to control cells. Although, the expression of N-cadherin or Vimentin was not detected in HPAC cells, which rarely express mesenchymal markers, downregulation of vimentin and upregulation of E-cadherin by GLRX3 knockdown was detected in the other pancreatic cancer cell line, CFPAC-1 (Additional file 1: Fig. S4). GLRX3-silenced HPAC cell lines also presented altered expression of Wnt pathway-related proteins (Fig. 5A). Wnt1, Wnt5a, Wnt7b, RhoA, and RhoB were downregulated in GLRX3 silenced cell lines. In contrast, β-catenin, Wnt4, and Wnt16 were upregulated in GLRX3- silenced cell lines compared to the control cells. Furthermore, EMT and the chemosensitivity related molecule, ABCG2, was reduced in the selected clones of shGLRX3 transformed cells.

Using the Pearson’s correlation coefficient and Kaplan-Meier survival estimate, we examined the correlation between GLRX3 mRNA and EMT-related gene expression in human pancreatic cancers in The Cancer Genome Atlas (TCGA) dataset. Figure 5B and C show the representative results of well-correlated TCGA data analyses with GLRX3 K/D cell line studies. A general tendency of negative correlation was observed between the expression levels of GLRX3 and Wnt4 (R = − 0.36, *p* < 0.0001). In contrast, Wnt7b and RhoA mRNA showed a positive correlation with GLRX3 mRNA expression (R = 0.36 and 0.2; *p* < 0.0001 and *p* = 0.0069, respectively). When the patients were divided into two groups based on mRNA expression levels, Wnt4 high expressers presented prolonged overall survival compared to low expressers, whereas RhoA and Wnt7b high expressers showed decreased overall survival compared to low expressers (All *p* < 0.05 by Log-rank test). The Pearson’s correlation coefficient between GLRX3 mRNA and other mRNAs, and the survival curves according to mRNA expression levels are demonstrated in Additional file 1: Figs. S2 and S3. These results suggest that GLRX3 expression altered the CSC features of pancreatic cancer by regulating the expression of EMT- and Wnt pathway-related molecules.

## Discussion

We identified novel secreted markers from pancreatic CSC-enriched spheres compared with adherent cells. The sphere culture method is a useful method for CSC enrichment using specific markers, in addition to the side population and sorting methods. In pancreatic cancer cells, GLRX3 was increased in spheres compared to adherent cells and was also increased in CD24+/CD44+/ESA+ cells compared to CD24−/CD44−/ESA- cells. GLRX3 was further expressed in human pancreatic tissues and blood samples. Serum GLRX3 expression was higher in patients with pancreatic cancer than in healthy controls. Moreover, we demonstrated for the first time that GLRX3 knockdown deprived pancreatic CSCs of their stemness properties in vitro and in vivo. GLRX3 silenced cell lines exhibited decreased proliferation, migration, and tumorigenesis. GLRX3 knockdown also reduced c-Met positive cells and altered the expression of stemness-related molecules.

CSCs are a subpopulation of cancer cells with high self-renewal capacity within tumors. Typically, CSCs constitute less than 5% of total tumor cells and are critical for cancer initiation, invasion, metastasis, and drug resistance [47, 48]. Recent studies have shown that cancer cells undergoing EMT share many properties with CSCs [49, 50]. Identification and characterization of CSCs in pancreatic cancer has been considered challenging as the features of CSCs overlap with those of normal stem cells. Marker detection specific to stemness, sphere-formation assays, and detection of side-population (SP) cells are general tools to identify CSCs [2]. Several CSC-specific markers including CD24, CD44, CXCR4, ABCG2, c-Met, and ALDH-1 have been reported in PDACs [2, 51]. Previously, we reported identification of subpopulations regarded as CSCs in pancreatic cancer, by sphere-formation assay or side-population cells as well as biomarkers related to pancreatic CSCs [11, 12, 44, 52]. However, multiple populations with the ability of tumor formation and self-renewal have been reported in pancreatic cancer. The CSCs population with each marker defined have been partially correlated with other CSC populations, but many reported markers have not been validated functionally. Therefore, to understand the function and relationship between markers, discovery of new marker candidates is required. Therefore, differentially expressed proteins in sphere formation cells were documented in our study by using proteomic methods.

Previously, Kanojia et al. isolated breast CSCs derived from spheres of HER2/Neu transgenic mice and identified the ferritin heavy chain 1 (FTH1) as a potential therapeutic target, by using LC-MS/MS [53]. Emmink et al. and Van Houdt et al. performed proteomic analysis of colorectal CSCs from spheres of primary tumors by using one-dimensional gel electrophoresis and nano LC-MS/MS and identified BIRC6 as a candidate target gene [54, 55]. For pancreatic CSCs, Zhu et al. identified glycoprotein markers in CD24+/CD44+ cells from a pancreatic cancer cell line as a prognostic marker. They also suggested proteins co-expressed with CD24 as a prognostic marker and therapeutic target by profiling frozen pancreatic CD24+ adenoma tissues [56, 57]. In our study, a total of 200 spots were differentially expressed between spheres and adherent cells by at least 2-fold, and 55 upregulated spots were identified using MALDI-TOF. Proteins known to be associated with cancer or CSCs such as HSP90AB1, ALDH, vimentin, and AKR were upregulated in spheres and their expression was confirmed by western blot. Among the upregulated proteins, GLRX3 was selected as a novel pancreatic CSC marker.

GLRX3 was first identified as a PKC θ-interacting protein in the early 2000s, and was studied in the context of stress response in immune cells and of hypertrophy in the heart [58–61]. Recently, GLRX3 expression has been correlated with human cancer. GLRX3 was found to be overexpressed in colon, lung, breast, and nasopharyngeal cancer; GLRX3 expression was also reported to have a positive correlation with patient survival [62–66]. Moreover, GLRX3 was also reported to be involved in tumor initiation and progression in various types of cancers via NF-κB signaling, [64, 67] Notch signaling, [68] stress-induced DNA damage responses, [69] and mTOR signaling [70]. There were no previous reports about the role of GLRX3 in pancreatic cancer; however, relatively high expression of GLRX3 mRNA expression was associated with the poor survival of PDAC patients in TCGA [71]. However, the functional role of GLRX3 in PDAC remains unknown. Furthermore, the relationship between CSCs and GLRX3 in human cancer is yet to be reported.

In our study, GLRX3 knockdown downregulated the Met/PI3K/AKT pathway in pancreatic cancer cells. GLRX3 was found to have a role in cell proliferation, metastasis, in vivo tumor formation, and tumor growth as well as in sphere formation and colony formation. GLRX3 knockdown reduced the proportion of c-Met positive cells, and decreased tumor formation in mouse models. When GLRX3 was downregulated using shRNA and siRNA in a pancreatic cancer cell line, c-Met and its downstream molecules such as PI3K and pAKT were decreased; however, c-Met knockdown did not affect GLRX3 expression. These results suggest that GLRX3 is an upstream regulator of c-Met. c-Met is the receptor tyrosine kinase for hepatocyte growth factor/scatter factor, and its activity was promoted by CD44 [46, 72]. CD44+/c-Met high cells were more tumorigenic compared to the low-c-Met expressing cells without CD44 in vivo [7]. Previous studies have reported that targeting the c-Met pathway overcomes chemo-resistance and stem cell signaling in pancreatic cancer [73, 74] and that Met inhibition induces chemosensitivity in gastric cancer stem cells [75]. Thus, GLRX3 may play a role in the CSCs of pancreatic cancer through the c-Met pathway.

Furthermore, GLRX3 knockdown altered the expression of several Wnt family members. Wnt1, Wnt5a, and Wnt7b were downregulated whereas Wnt4 and Wnt16 were upregulated in GLRX3-silenced cell lines. Wnt pathways have been divided into canonical (β-catenin dependent) or non-canonical (β-catenin independent) pathways [76]. Wnt proteins can be roughly grouped as canonical (Wnt1, Wnt2, Wnt3, Wnt3a, Wnt8a, Wnt8b, Wnt10a, Wnt10b), and noncanonical (Wnt4, Wnt5a, Wnt5b, Wnt7a, Wnt7b, Wnt11) proteins [77]. In our results, RhoA, one of the key molecules of the non-canonical pathway, was downregulated whereas β-catenin was upregulated. This finding suggested that the effect of GLRX3 knock-down on Wnt protein alterations could play a role via the noncanonical Wnt pathway. Non-canonical Wnt signaling in pancreatic cancer potentiated of chemoresistance and metastasis through EMT and cancer stemness [78]. In previous reports, Wnt5a induce EMT and potentiate metastasis across multiple cancer types through non-canonical mechanisms [79] and also mediate gemcitabine resistance in PDAC via upregulation of ABCG2 [80]. In another report, Wnt5a promoted cell migration via the PI3K/AKT/GSK3b/RhoA signaling pathway in gastric cancer [81]. High expression of Wnt7a associated with poor prognosis and metastasis in PADC [82] and could predict metastasis of colorectal **cancer** via EMT and poor prognosis [83]. In our data, GLRX was considered to regulate the cancer stem cell phenotype via non-canonical Wnt signaling pathways associated with RhoA and ABCG2 via Wnt5a and Wnt7b. It is postulated that alterations reported in other Wnt proteins may be associated with PDAC in different or identical pathways, but further studies are needed to make it conclusive.

Epithelial-mesenchymal transition (EMT) is an important biological process in the progression of primary tumors toward metastasis and drug resistance in solid tumors including pancreatic cancer. In EMT, epithelial adhesion molecules such as E-cadherin and/or cytokeratin are decreased and mesenchymal markers such as N-cadherin, vimentin and/or fibronectin are induced. A previous report indicated that GLRX3 is involved in epithelial-to-mesenchymal transition (EMT) in breast cancer, [84]; therefore, we examined the changes in EMT marker by GLRX3 knockdown in pancreatic cancer cells. GLRX3 was partially involved in the EMT process of pancreatic cancer. In the present study, the epithelial marker E-cadherin was induced, and the mesenchymal marker Vimentin was reduced in GLRX3 knockdown cells. Further, GLRX3 knockdown reduced the level of ABCG2, EMT, and chemo-resistance related proteins in pancreatic cancer cells. ABCG2 is frequently reported as a chemoresistance, as well as cancer stem cell-related marker [85, 86]. These results suggested that GLRX3 is partially involved in the EMT process and that CSCs and EMT cells are linked with the phenotypic and molecular changes.

In our study, information from TCGA was used to validate the results of GLRX3 knockdown studies in pancreatic cell lines. The protein expression of GLRX3 and c-MET presented meaningful correlation in the cell line study, and the mRNA expression of GLRX3 and Met showed a significant positive correlation with Pearson’s correlation value, as observed using the TCGA database. Survival analysis according to the mRNA expression level of GLRX3 and Met showed similar significant differences between the high and low expression groups. Further, the results of protein expression related to stemness and EMT were further verified using TCGA data; these results added reliability to the results of our experiment.

Although GLRX3 is a potential secretory protein, there was no report regarding GLRX3 detection in patient blood or in cell culture medium. In our study, GLRX3 overexpression was consistently detected in the blood of patients with pancreatic cancer and in the media of cultured cells as well as in the tissues of patients with pancreatic cancer. In western blot analysis, GLRX3 protein expression was increased in the plasma of patients with pancreatic cancer than in the plasma of healthy persons or in patients with chronic pancreatitis. Furthermore, using a commercial ELISA kit, GLRX3 was found to be highly secreted into the serum of patients with pancreatic cancer than in the healthy controls. As a diagnostic marker, GLRX3 showed sensitivity similar to CA19–9 in our results. When GLRX3 and CA19–9 were combined, the sensitivity was increased to 98.3% with 100% of specificity and 0.99 of AUC. These results are significantly superior to those of GLRX3 or CA19–9 alone. These data thus indicate that GLRX3 can be a potential diagnostic biomarker for pancreatic cancer, alone or in combination with CA19–9. Furthermore, high serum GLRX3 levels in ELISA were significantly associated with poor DFS after surgery. This result suggests that GLRX3 may be associated with tumor recurrence after surgical treatment. However, since the number of healthy controls included in the ELISA analysis is small, caution is needed in interpreting the diagnostic significance of serum levels of GLRX3. To verify the diagnostic ability of the biomarkers, especially the specificity, the number of heathy controls of only 10 is not a sufficient number for statistical analysis. It should be verified in a larger number of healthy controls, however, it was not practically possible in our study. Further studies need to be conducted on a larger number of patient samples to verify our results.

In conclusion, our study describes the secretory proteomic profile for pancreatic CSCs including already known markers and a novel marker, GLRX3. The level of GLRX3 expression was elevated in cancer cell lines and in the tissues and blood from patients with pancreatic cancer. In a functional study, GLRX3 was involved in cancer cell proliferation, migration, invasion, tumorigenesis, and maintenance of CSC properties. GLRX3 thus seems to regulate the CSC phenotype through c-Met and Wnt signaling. These results suggest that GLRX3 is a new potential biomarker for pancreatic cancer, as well as a therapeutic target for pancreatic CSCs.

## Supplementary Information

**Additional file 1: Fig. S1.** 2-DE gel image of secretomes from adherent cells and spheres. **Fig. S2.** Pearson’s correlation between the expression of GLRX3 and various genes in TCGA data. **Fig. S3.** Survival analysis according to the mRNA expression of various genes in TCGA data. **Fig. S4.** EMT-related signaling in CFPAC-1 GLRX3 k/d clones. **Table S1.** Upregulated secretory proteins in spheres compared to those in adherent pancreatic cancer cells based on 2DE-PAGE and MALDI-TOF results. **Table S2.** Characteristics of patients with PDAC according to GLRX3 expression by IHC.,**Table S3.** Plasma samples used for validation of western blot analysis. **Table S4.** Demographics and clinical characteristics of the PDAC patient cohort for ELISA.

## Data Availability

The data presented in this study are available on request from the corresponding author. The data are not publicly available due to the personal information protection policies of the institutional review board.
