# Supplementary material for: GLRX3, a novel cancer stem cell-related secretory biomarker of pancreatic ductal adenocarcinoma
Source: BMC Cancer. 2021 Nov 18;21:1241. doi: 10.1186/s12885-021-08898-y (PMC8603516; doi:10.1186/s12885-021-08898-y)
Supplement: Supplementary file 1 — Additional file 1: Fig. S1. 2-DE gel image of secretomes from adherent cells and spheres. Fig. S2. Pearson’s correlation between the expression of GLRX3 and various genes in TCGA data. Fig. S3. Survival analysis according to the mRNA expression of various genes in TCGA data. Fig. S4. EMT-related signaling in CFPAC-1 GLRX3 k/d clones. Table S1. Upregulated secretory proteins in spheres compared to those in adherent pancreatic cancer cells based on 2DE-PAGE and MALDI-TOF results. Table S2. Characteristics of patients with PDAC according to GLRX3 expression by IHC.,Table S3. Plasma samples used for validation of western blot analysis. Table S4. Demographics and clinical characteristics of the PDAC patient cohort for ELISA. [file 12885_2021_8898_MOESM1_ESM.docx]

**Supplementary Materials**

Figure S1: 2-DE gel image of secretomes from adherent cells and spheres.

Figure S2: Pearson’s correlation between the expression of GLRX3 and various genes in TCGA data.

Figure S3: Survival analysis according to the mRNA expression of various genes in TCGA data.,

Figure S4: EMT-related signaling in CFPAC-1 GLRX3 k/d clones.

Table S1: Upregulated secretory proteins in spheres compared to those in adherent pancreatic cancer cells based on 2DE-PAGE and MALDI-TOF results.

Table S2: Characteristics of patients with PDAC according to GLRX3 expression by IHC.,

Table S3: Plasma samples used for validation of western blot analysis.

Table S4. Demographics and clinical characteristics of the PDAC patient cohort for ELISA.

**Supplementary figures**

**
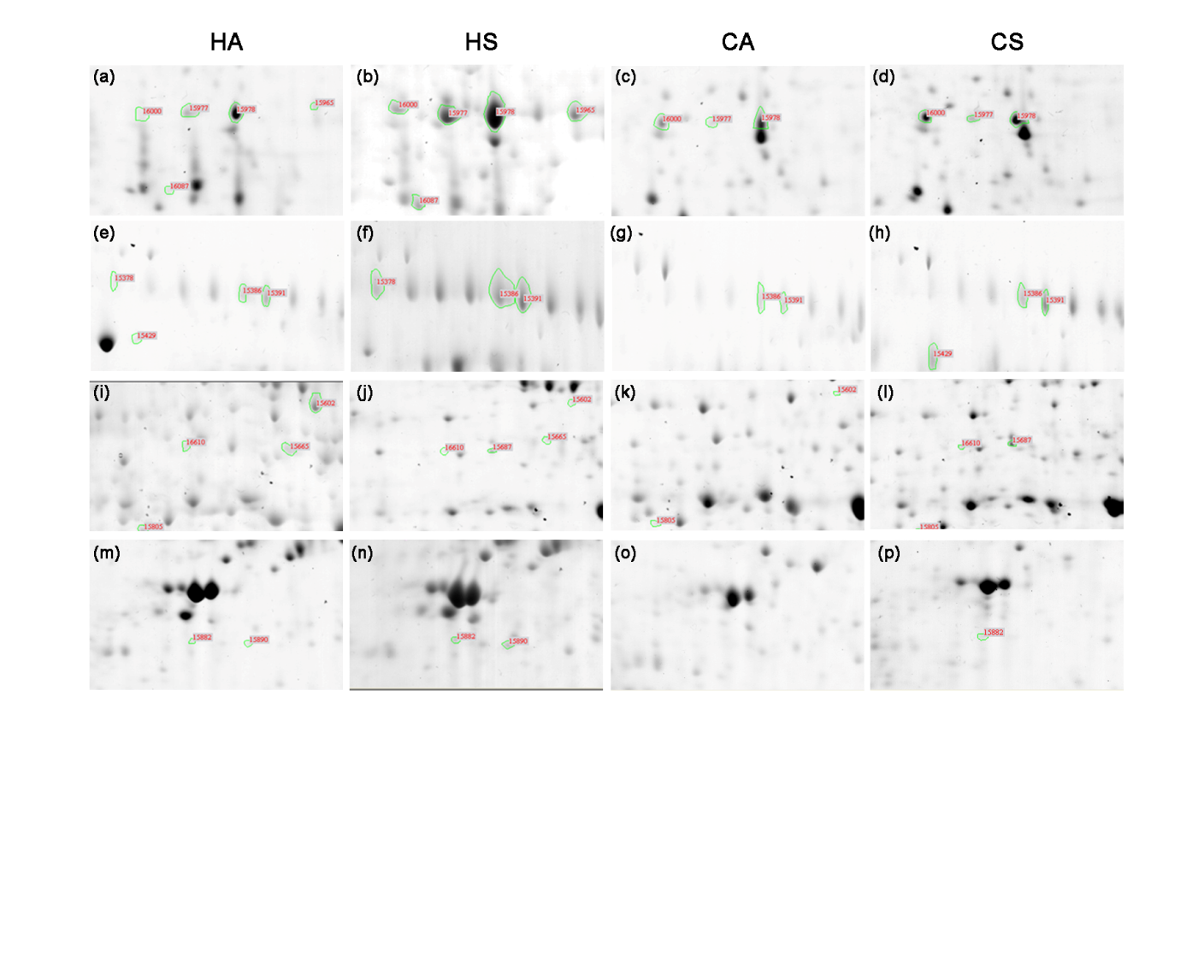
**

**Figure S1.** 2-DE gel image of secretomes from adherent cells and spheres: adherent HPAC cells (HA), spheres of HPAC (HS), adherent CAPAN-1 cells (CA), and spheres of CAPAN-1 (CS). Five representative differentially expressed spots were highlighted in the adherent cells and spheres of the HPAC 2DE gel images. (a–d) AKR1B1; spot 15977/15978/16000, (e–h) HSP90AB1; spot 15391, (i–j) ALDI; spot 16610, Vimentin; spot 15687, (m–p) GLRX3; spot 15890.

**Figure S2.** Pearson’s correlation between the mRNA expression of GLRX3 (Y-axis) and various genes (X-axis) in TCGA data: mRNA expression levels of various genes in pancreatic cancer tissues were downloaded from TCGA by using ISB Cancer Genomics Cloud. Outlier samples were removed by clipping the top and bottom deciles of each distribution. The correlations between GLRX3 and representative gene mRNA expression are illustrated as scatter plots where each dot represents a single cancer tissue sample. Pearson’s correlation value (R) and the p value are indicated.

 **Figure S3.** Survival analysis according to the mRNA expression of various genes in TCGA data: The mRNA expression of various genes and cancer patients’ clinical data were derived from TCGA. Patients were divided into high (blue color) or low (red color) expression groups, using maximally selected rank statistics. Survival of patients was visualized using Kaplan–Meier plots. Log-rank p values between the groups are shown in each plot.

**Figure S4.** EMT-related signaling in GLRX3 knockdown clones of CFPAC-1: (A) Semi-quantitative RT-PCR for Vimentin, E-cadherin, and Beta-catenin levels in control CFPAC-1 cells (F8 and A8) and GLRX3 knockdown clones (B10 and F5). E-cadherin and beta-catenin mRNA was overexpressed in GLRX3 knockdown clones; (B) Expression of EMT-related proteins in GLRX3 knockdown CFPAC-1 cells. Silenced cell lines (B10 and F5) presented altered expression of EMT-related proteins. E-cadherin and beta-catenin were upregulated and Vimentin was downregulated compared to the control cell line (F8 and A8).

**Supplementary Table S1.** Upregulated secretory proteins in cell spheres compared to adherent pancreatic cancer cells, based on 2DE-PAGE and MALDI-TOF

| **Group ID** | **S/A* ratio** | **Protein name** | **Accession no. (SWISS/Prot)** | **Score^†^** | **Matched peptide** |
| --- | --- | --- | --- | --- | --- |
| 15378 | 4.1 | Hypothetical protein | gi\|30268237 | 84 | 16 |
| 15386 | 2.5 | Metalloprotease 1 precursor | gi\|41352061 | 69 | 14 |
| 15391 | 2.7 | Heat shock 90kDa protein 1, beta | gi\|20149594 | 67 | 14 |
| 15413 | 2.7 | Heat shock 70kDa protein 5 | gi\|16507237 | 70 | 13 |
| 15429 | 4.3 | Hypothetical protein LOC55471 isoform 3 | gi\|145701028 | 67 | 9 |
| 15436 | 9.2 | 1-acylglycerol-3-phosphate O-acyltransferase 4 | gi\|56203587 | 67 | 6 |
| 15445 | 2.7 | Keratin, type II cytoskeletal 1 | gi\|1346343 | 63 | 12 |
| 15470 | 2.3 | Glutaredoxin | gi\|4504025 | 68 | 5 |
| 15538 | 2.0 | Tumor rejection antigen | gi\|61656607 | 80 | 21 |
| 15602 | 5.2 | HSP90AB1 protein | gi\|39644662 | 73 | 14 |
| 15619 | 2.8 | Hypothetical protein LOC55471 isoform 1 | gi\|21396487 | 75 | 11 |
| 15665 | 2.3 | Uunnamed protein product | gi\|31543 | 73 | 13 |
| 15680 | 2.2 | TUBB3 protein | gi\|38014278 | 158 | 21 |
| 15687 | 2.3 | Vimentin | gi\|62414289 | 73 | 13 |
| 15737 | 4.3 | Dynein, axonemal, heavy chain 3 | gi\|24308169 | 74 | 40 |
| 15745 | 3.0 | Plasma kallikrein | gi\|125184 | 67 | 12 |
| 15792 | 2.3 | Glutaredoxin | gi\|4504025 | 56 | 5 |
| 15805 | 2.0 | hCG2042304 | gi\|119586807 | 53 | 5 |
| 15821 | 3.3 | Hypothetical protein FLJ10808 isoform | gi\|34304594 | 72 | 12 |
| 15824 | 9.6 | Mixture 1(GLRX3 protein + activator of heat shock 90kDa protein ) | gi\|48257132 + gi\|6912280 | 160 |  |
| 15871 | 3.3 | Erythrocyte 26 S protease subunit 12 | gi\|998688 | 70 | 9 |
| 15882 | 6.2 | RPS6KA4 protein | gi\|28839796 | 66 | 12 |
| 15890 | 2.1 | GLRX3 protein | gi\|48257132 | 99 | 14 |
| 15907 | 3.2 | PREDICTED: hypothetical protein XP_002342056 | gi\|239741018 | 68 | 13 |
| 15965 | 11.1 | Chain A, Human 3alpha-Hsd Type 3 | gi\|21465695 | 119 | 17 |
| 15970 | 2.0 | Glyceraldehyde-3-phosphate dehydrogenase | gi\|31645 | 97 | 18 |
| 15977 | 3.8 | Aldo-keto reductase family 1, member B1 | gi\|4502049 | 80 | 12 |
| 15978 | 3.4 | Aldo-keto reductase family 1 | gi\|4502049 | 94 | 14 |
| 16000 | 2.3 | Chain A, Human Aldose Reductase Mutant V47i | gi\|171848760 | 76 | 12 |
| 16026 | 2.5 | Formin 2-like protein | gi\|8118090 | 67 | 10 |
| 16051 | 17.0 | Hypothetical protein LOC146705 | gi\|21389577 | 67 | 9 |
| 16084 | 4.3 | Mixture 1( EF-hand domain family+cathepsin D preproprotein ) | gi\|20149675 + gi\|4503143 | 157 | 25 |
| 16087 | 4.4 | Nucleoside phosphorylase | gi\|157168362 | 94 | 15 |
| 16095 | 3.7 | Cathepsin D preproprotein | gi\|4503143 | 100 | 15 |
| 16139 | 2.1 | Unnamed protein product | gi\|158261815 | 69 | 19 |
| 16159 | 2.6 | TPI1 protein | gi\|47682755 | 86 | 11 |
| 16193 | 2.5 | Heat shock protein 27 | gi\|662841 | 69 | 8 |
| 16246 | 2.2 | Chain A, Crystal Structure Of Siderocalin | gi\|60593959 | 98 | 11 |
| 16268 | 2.1 | Biliverdin-IX beta reductase isozyme I | gi\|544759 | 103 | 13 |
| 16276 | 2.2 | Keratin 9 | gi\|453155 | 113 | 16 |
| 16288 | 2.0 | Unamed protein product | gi\|14042653 | 58 | 8 |
| 16337 | 2.0 | Tetraspanin 18, isoform CRA_a | gi\|119588464 | 66 | 8 |
| 16432 | 2.2 | Unnamed protein product | gi\|158259341 | 60 | 14 |
| 16483 | 2.2 | PREDICTED: hypothetical protein isoform 1 | gi\|114580754 | 66 | 11 |
| 16490 | 2.1 | PREDICTED: similar to major histocompatibility complex | gi\|239740855 | 67 | 8 |
| 16528 | 2.5 | Unnamed protein product | gi\|16549206 | 60 | 9 |
| 16610 | 2.9 | Aldehyde dehydrogenase | gi\|178375 | 63 | 11 |
| 16625 | 5.6 | Serine (or cysteine) proteinase inhibitor, clade B | gi\|13489087 | 85 | 12 |
| 16629 | 9.4 | A-kinase anchoring protein AKAP350 | gi\|4558862 | 56 | 26 |
| 16640 | 2.4 | Unnamed protein product | gi\|21752882 | 68 | 13 |
| 16643 | 5.3 | Spectrin repeat containing, nuclear envelope 1 | gi\|119568120 | 72 | 21 |
| 16650 | 2.3 | hCG2015069 | gi\|119617987 | 71 | 8 |
| 16663 | 2.7 | Chain A, Crystal Structure Of Siderocalin | gi\|60593959 | 98 | 11 |
| 16670 | 3.1 | Chain A | gi\|114794585 | 66 | 9 |
| 16676 | 3.6 | Transferrin | gi\|115394517 | 57 | 14 |

* Enriched secretory proteins in spheres, compared with adherent cells. S/A ratios indicated expression level of protein in spheres, compared to that in adherent cells.

^†^ Ion scores greater than the threshold with a significant p-value were presented according to the Mascot search results.

**Supplementary Table S2.** Characteristics of patients with PDAC according to GLRX3 expression by IHC

|  | Total n = 32 | GRLX3 (-)  n = 12 | GLRX3 (+)  n = 20 | P value* |
| --- | --- | --- | --- | --- |
| Age at diagnosis, Mean (SD) | 60.8 (±9.7) | 62.6 (±10.0) | 59.7 (±9.6) | 0.430 |
| Gender (%) |  |  |  | 0.815 |
| Female | 26 (81.2%) | 10 (83.3%) | 16 (20.0%) |  |
| Male | 6 (18.8%) | 2 (16.7%) | 4 (20.0%) |  |
| CA 19-9, IU/mL, mean (SD) | 276.6 (±302.0) | 247.1 (±369.5) | 294.2 (±262.4) | 0.703 |
| pT stage (%) |  |  |  | 0.190 |
| T2 | 1 (3.1%) | 1 (8.3%) | 0 (0.0%) |  |
| T3 | 31 (96.9%) | 11 (91.7%) | 20 (100.0%) |  |
| pN stage (%) |  |  |  | 0.258 |
| N0 | 20 (62.5%) | 6 (50.0%) | 14 (70.0%) |  |
| N1 | 12 (37.5%) | 6 (50.0%) | 6 (30.0%) |  |
| Stage (%) |  |  |  | 0.200 |
| I | 1 (3.1%) | 1 (8.3%) | 0 (0%) |  |
| II | 19 (59.4%) | 5 (41.7%) | 14 (70.0%) |  |
| III | 12 (37.5%) | 6 (50.0%) | 6 (30.0%) |  |
| Differentiation (%) |  |  |  | 0.162 |
| Moderate | 23 (71.9%) | 8 (66.7%) | 15 (75.0%) |  |
| Poor | 7 (21.9%) | 2 (16.7%) | 5 (25.0%) |  |
| Undifferentiated | 2 (6.3%) | 2 (16.7%) | 0 (0%) |  |
| Resection margin (%) |  |  |  | 0.581 |
| R0 | 25 (87.1%) | 10 (83.3%) | 15 (75.0%) |  |
| R1 | 7 (21.9%) | 2 (16.7%) | 5 (25.0%) |  |
| Recurrence after surgery (%) |  |  |  | 0.403 |
| Yes | 13 (40.6%) | 6 (50.0%) | 7 (35.0%) |  |
| No | 19 (59.4%) | 6 (50.0%) | 13 (65.0%) |  |
| Median Survival, months (95% CI) |  |  |  |  |
| Disease free survival | 13.0 (2.1-23.9) | 15.4 (0.0-32.7) | 9.0 (1.7-16.8) | 0.553 |
| Overall survival | 17.6 (9.2-26.0) | 21.5 (15.8-27.2) | 13.9 (8.0-19.8) | 0.855 |

*Abbreviations: PDAC, pancreatic ductal adenocarcinoma; IHC, immunohistochemistry; SD, standard deviation; CI, confidence interval.*
* P-values were calculated using the χ­2 and Fisher’s exact tests for categorical data, Student’s *t*-tests, and Mann-Whitney U tests for continuous variables and Kaplan-Meier analysis with a Log-rank test for survival analysis.

**Supplementary Table S3.** Plasma samples used for validation of western blot results

|  | **Cases (n)** | **Gender** | **Age range (median)** |
| --- | --- | --- | --- |
| **NL** | **5** | **Male = 3** | **29-33 (33)** |
|  |  | **Female = 2** |  |
| **CP** | **5** | **Male = 4** | **30-63 (47)** |
|  |  | **Female = 1** |  |
| **PC** | **20** | **Male = 9** | **39-78 (60)** |
|  |  | **Female = 11** |  |

| **Disease** | **Sample no.** | **Sex** | **Age** | **Disease** | **Sample no.** | **Sex** | **Age** |
| --- | --- | --- | --- | --- | --- | --- | --- |
| **Normal** | **1** | **M** | **34** | **Pancreatic cancer** | **6** | **M** | **55** |
|  | **2** | **F** | **30** |  | **7** | **F** | **39** |
|  | **3** | **M** | **29** |  | **8** | **M** | **66** |
|  | **4** | **M** | **33** |  | **9** | **M** | **70** |
|  | **5** | **F** | **33** |  | **10** | **M** | **53** |
| **Chronic pancreatitis** | **1** | **F** | **52** |  | **11** | **F** | **75** |
|  | **2** | **M** | **41** |  | **12** | **M** | **66** |
|  | **3** | **M** | **63** |  | **13** | **F** | **53** |
|  | **4** | **M** | **47** |  | **14** | **F** | **52** |
|  | **5** | **M** | **30** |  | **15** | **M** | **67** |
| **Pancreatic cancer** | **1** | **M** | **45** |  | **16** | **F** | **60** |
|  | **2** | **F** | **78** |  | **17** | **M** | **57** |
|  | **3** | **F** | **60** |  | **18** | **F** | **53** |
|  | **4** | **M** | **51** |  | **19** | **F** | **67** |
|  | **5** | **F** | **67** |  | **20** | **F** | **66** |

**Supplementary Table S4.** Demographics and clinical characteristics of the PDAC patients’ cohort for ELISA

|  |  | Total N=60 | % or SD or 95%CI |
| --- | --- | --- | --- |
| Sex (%) | Male | 40 | 66.7% |
|  | Female | 20 | 33.3% |
| Age, mean (SD) | | 62 | 13 |
| Stage (%) | II | 23 | 38.3% |
|  | III | 11 | 18.3% |
|  | IV | 26 | 43.3% |
| CA19-9, IU/L, mean (SD) | | 2680.66 | 5013.54 |
| GLRX3, ng/mL, mean (SD) | | 101.41 | 92.03 |
| Overall survival, month, median (95%CI) | | 13.5 | 9.4-17.6 |
| Surgical resection (%) | Yes | 23 | 38.3% |
|  | No | 37 | 61.7% |
| Recurrence after surgery* (%) | yes | 18 | 90.00% |
|  | no | 2 | 10.0% |
| Disease free survival, month, median (95%CI) | | 9.1 | 4.2-14.0 |
| Overall survival, month, median (95%CI) | | 22.0 | 18.5-25.5 |
| Palliative chemotherapy (%) | Yes | 51 | 85.0% |
|  | No | 9 | 15.0% |
| Chemotherapy regimens (%) | FOLFIRINOX | 3 | 5.9% |
|  | CCRT | 14 | 27.5% |
|  | GT | 19 | 37.3% |
|  | GP | 13 | 25.5% |
|  | Xeloda | 2 | 3.9% |
| Progression after chemotherapy** (%) | | 47 | 100% |
| Progress free survival, month, median (95%CI) | | 3.0 | 1.4-4.5 |
| Overall survival, month, median (95%CI) | | 7.7 | 4.8-10.6 |

*Abbreviations: PDAC, pancreatic ductal adenocarcinoma; ELISA, enzyme-linked immunosorbent assay; SD, standard deviation; CI, confidence interval.** Three cases were not evaluated due to follow-up loss.
** Four cases were not evaluated due to follow-up loss.
